# Evaluation of the Immediate Effect of Auricular Acupuncture on Pain and Electromyographic Activity of the Upper Trapezius Muscle in Patients with Nonspecific Neck Pain: A Randomized, Single-Blinded, Sham-Controlled, Crossover Study

**DOI:** 10.1155/2015/523851

**Published:** 2015-09-16

**Authors:** Andréia Cristina de Oliveira Silva, Daniela Aparecida Biasotto-Gonzalez, Douglas Meira dos Santos, Nivea Cristina De Melo, Cid André Fidelis de Paula Gomes, César Ferreira Amorim, Fabiano Politti

**Affiliations:** ^1^Postgraduate Program in Rehabilitation Sciences, Universidade Nove de Julho (UNINOVE), Avenida Dr. Adolfo Pinto 1w09, Água Branca, 05001-100 São Paulo, SP, Brazil; ^2^Department of Physical Therapy, Universidade Nove de Julho (UNINOVE), Avenida Dr. Adolfo Pinto 109, Água Branca, 05001-100 São Paulo, SP, Brazil; ^3^Physical Therapy Program, Universidade Cidade de São Paulo (UNICID), Rua Cesário Galeno, Tatuapé, 448/475 São Paulo, SP, Brazil

## Abstract

*Background*. The aim of the present study was to assess the immediate effects of auricular acupuncture (AA) on the electromyographic (EMG) activity of the upper trapezius muscle and pain in nonspecific neck pain (NS-NP) patients. Twelve patients with NS-NP (NS-NP group) and 12 healthy subjects (HS Group) were enrolled in a randomized, single-blinded, crossover study. Each subject received a single session of AA and sham AA (SAA). Surface EMG activity was measured in the upper trapezius muscle at different “step contractions” of isometric shoulder elevation (15%, 20%, 25%, and 30% MVC). The outcome measure in patients with NS-NP was based on the numerical pain rating scale (NRS). AA treatment led to a significant decrease in EMG activity in both groups (NS-NP group: *p *= 0.0001; HS group: *p* < 0.0001—ANOVA test). This was not the case for the SAA treatment (NS-NP group: *p* = 0.71; HS group: *p* < 0.54). Significant decreases (*p* < 0.001) in the NRS were found for both treatments (AA and SAA). This study demonstrated the immediate effect of auricular acupuncture on the electromyographic activity of the upper trapezius muscle but the effect of this intervention on pain symptoms in patients with nonspecific neck pain was inconclusive.

## 1. Introduction

Cervical pain can cause great personal suffering, incapacity, a lower quality of life, and reduced productivity, as well as creating high socioeconomic costs for patients and society as a whole [1–3]. For most sufferers, its course involves remission and exacerbation rather than chronic or complete resolution [2]. However, one specific cause cannot be identified and thus the term nonspecific neck pain (NS-NP) has been commonly used as its classification [4].

The main clinical characteristics of patients with NS-NP are morphofunctional abnormalities in the neck [5], usually accompanied by pain in the head and/or neck, which may or may not irradiate to the upper limbs, as well as abnormal sensitivity and rigidity in the shoulder girdle muscles [6].

With regard to muscular abnormalities, surface electromyography (EMG) has previously demonstrated that individuals with cervicalgia exhibit a different pattern of activity than healthy individuals [7–9]. In addition, there is strong evidence that the motor control of the upper trapezius muscle of patients with NS-NP is directly affected by cervical pain during isometric activities, when compared with healthy control individuals [8]. A number of authors have explored the possibility that an abnormality in cervical motor control could contribute to the persistence of pain in this region due to factors that perpetuate a mechanical nociceptive mechanism in cervical structures, as well as muscle fatigue, which is inherent to these patients [10–12].

Several techniques of treating NS-NP have been shown to be effective in terms of achieving a clinical improvement in patients, including muscular massage, stretching, specific therapeutic exercises, scapular movements, physiotherapeutic resources (electrothermal), and acupuncture [9, 13–16]. However, there is still no consensus on the best form of treating NS-NP.

Although the use of acupuncture to treat NS-NP [17] has been accepted and recommended, there remain arguments about the effects of this treatment, the form of therapeutic approach (different quantities of acupoints and the number of sessions) [17, 18], and the treatment technique to be used (systemic, auricular, and cranial acupuncture) [17–20]. These arguments hinder the selection of this resource as a clinical approach for NS-NP, as well as a clear understanding of its physiological mechanisms.

With regard to the different approaches of acupuncture, the results presented in a recent literature review suggest that auricular acupuncture (AA) can be used as an adjunct therapy for pain management, thereby reducing the use of analgesics and minimizing the potential adverse effects and tolerance [21].

The theory and body mapping protocol proposed by the French physician Paul Nogier indicates that the needle stimulus in the specific external auricular pavilion can have a reflexive influence on specific regions of the body [22, 23]. Due to these relationships, needling and/or stimulating one or more ear acupuncture points can be performed to treat specific organ functions.

Only one previous study [24] has demonstrated the real possibilities of the action of AA on the activity of the upper trapezius muscle in healthy individuals. No studies were found that addressed the possible clinical benefits of the treatment proposed by Nogier for NS-NP. Thus, since there is evidence of a relationship between the upper trapezius muscle and specific acupoints in the ear [24], it is possible that this relationship becomes more evident in individuals with NS-NP.

In clinical practice, confirmation that the insertion of needles in specific points of the ear can act directly on the upper trapezius muscle of individuals with NS-NP could contribute to the following: (i) a standardization of the points to be used as stimuli for the treatment of abnormalities caused in this muscle by mechanical injuries, myofacial tension (with or without trigger-points), and systemic diseases such as fibromyalgia; (ii) an improvement in the manner of estimating the effects of interventions; (iii) simplifying the use of this resource; and (iv) stimulating its use in isolation or concomitantly with other treatment methods.

The aim of this study was to assess the immediate effects of auricular acupuncture on the electromyographic activity of the upper trapezius muscle and pain in patients with nonspecific neck pain.

## 2. Methods

### 2.1. Subjects

Twelve NS-NP patients (NS-NP group) and 12 health subjects (HS group) participated in this study (Table 1). The sample size was calculated considering *α* = 0.05 (5% chance of type 1 error) and 1 − *β* = 0.99 (% of power of the sample) and data on the amplitude of the EMG signal in the study by Chou et al. [25]. The values were those described in the period prior to acupuncture (21.3 ± 9.5 *μ*V) and after three minutes of needle manipulation in the acupoints TE-5 and LI-11 (9.5 ± 3.5 *μ*V).

Although this study used traditional acupuncture, it was selected because it was the only study found that provided EMG evidence of acupuncture having a significant effect on muscle activity.

The inclusion criteria for the NS-NP group were the following: score range of 15–24 on the Neck Disability Index (adapted and validated for the Brazilian population), which specifically evaluates neck pain and disability [26]; history of neck pain for a period of more than three months; restricted active or passive neck movement in at least one direction; and score range of 3–6 points on an 11-point (range: 0 to 10) numerical rating scale for perceived pain intensity (NRS) [27]. The NRS has been translated and cross-culturally adapted for the Brazilian population [28]. To HS group, the inclusion criteria were no positive signs of cervical spine or scapular dysfunction during the physical examination and no self-reported history of neck pain.

The exclusion criteria adopted in this study were the following: use of analgesic, muscle relaxant, psychotropic agent, or anti-inflammatory agent in the previous three days; chronic neck pain resulting from a traumatic incident; chronic musculoskeletal condition (e.g., muscular disorder, polyarthritis); history of neurological disorders (i.e., irradiated pain) or neck surgery; systemic disease; connective tissue disorder; body mass index > 25 kg/m; nontolerance of needles; current pregnancy; medical diagnosis of fibromyalgia; and having undergone physical therapy, massage, or acupuncture in the previous two weeks.

The present study was approved by the Human Research Ethics Committee of the Universidade Nove de Julho, under protocol number 525.849/2012. All participants/guardians were properly informed regarding the objectives and procedures and signed a statement of informed consent prior to testing.

### 2.2. Randomisation and Blinding

**Figure 1:**
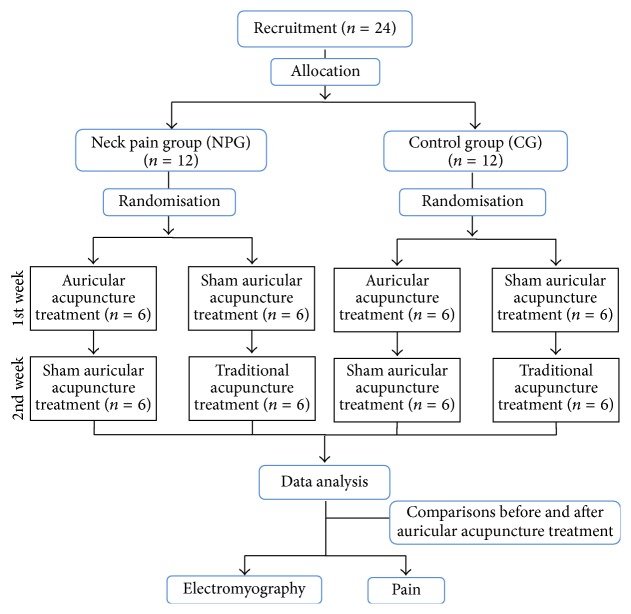
Flowchart of the study.

A single-blinded, randomized, sham-controlled, crossover, clinical trial was conducted. The crossover design was used to exclude the potential interference of individual differences.  Figure 1 displays the flowchart of the study.

Each volunteer received two forms of acupuncture in random order: a single session of AA (Nogier method) [22, 23] and sham AA (SAA). To eliminate carry-over treatment effects, a one-week wash-out period between the treatments was respected. Randomisation in each group was performed by lots using opaque envelopes containing either the letter “Y,” corresponding to AA, or “X,” corresponding to SAA. The participants in each group will be distributed into two subgroups of six individuals based on the initial treatment (AA and SAA). When one of the subgroups (AA or SAA) was completed, the order of consecutive arrival of the volunteers was used until all individuals have been allocated.

The volunteers and researchers responsible for analyzing the data were blinded to the type of treatment and not informed that one of the two treatments is a sham procedure. After the experiment, all participants were informed that they received treatment with AA and SAA.

### 2.3. Electromyography

The sEMG signal was recorded in the dominant upper trapezius muscle in the CG and on the side with the greatest self-reported pain in the NPG. EMG signals were obtained using a 16-channel module (EMG System do Brasil, Ltda.), with a band pass filter with cut-off frequencies of 20 to 500 Hz, an amplifier gain of 1000, and a common rejection mode ratio > 120 dB. All data was acquired and processed using a 16-bit analog to digital converter, with a sampling frequency of 2 kHz.

The bipolar surface circular electrodes (Ag/AgCl, Medical Trace) with 10 mm diameter were used for the surface recording of EMG with a center-to-center distance of 20 mm. Before electrode placement, the skin will be cleaned using abrasive paste. The electrode was positioned 2 cm lateral to the midpoint of the line between the C7 spinous process and the acromion [29].

### 2.4. Auricular Acupuncture

**Figure 2:**
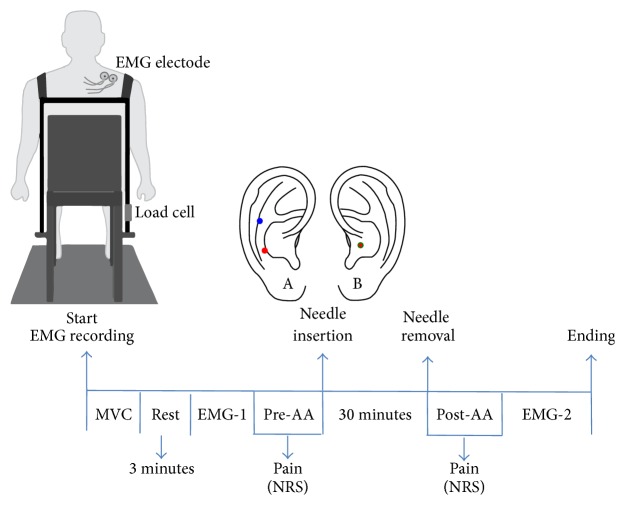
Flow sequence diagram of data recording. A: auricular acupuncture (Nogier method). B: sham auricular acupuncture.

In the AA treatment, sterile acupuncture needles measuring 0.25 × 13 mm (Suzhou Huanqiu Acupuncture Medical Appliance Co., Ltd.) were inserted on the ear at the points corresponding to the scapular waist, located in the sixth of seven spaces contained between the posterior folds of the antitragus (in the region of its junction with the antihelix and the second depression located on the antihelix), and to the shoulder, located approximately 3 mm above the furrow which separates the antihelix from the antitragus as indicated in Figure 2 [22–24].

In the SAA treatment, the needles were inserted on the shell of the ear (Figure 2), especially that this region does not present any somatotopic relationship to the shoulder and the scapular waist [23]. A physical therapist certified in the Nogier method of auricular acupuncture with 15 years of experience performed the auricular treatment. The needle insertion sites were previously cleaned with alcohol.

After the experiment, all participants in the NGP will be sent for physical therapy at the rehabilitation clinic of the university.

### 2.5. Procedures

Figure 2 displays the sequence of the experiment. The evaluations were performed with the patient sitting comfortably in a chair with both feet flat on the floor, hips and knees flexed at 90°, buttocks positioned against the back of the chair, and treated shoulder unclothed.

Disposable electrodes were attached for the collection of the sEMG signal in the upper trapezius muscle treated with acupuncture. Straps were hung from the shoulders and connected in front and back by another strap with Velcro to allow adjustments to the chest size of each individual. The ipsilateral strap to the shoulder on which the sEMG signal was read was attached to a load cell (EMG System do Brasil, Ltda.) connected to a support attached to the chair (Figure 2). The strap on the contralateral shoulder was attached directly to the chair. The straps were individually adjusted and the volunteer was instructed to raise the shoulder to be analyzed in maximum voluntary contraction (MVC) for 5 s with a three-minute rest interval between readings. The maximum value in Newton was considered the MVC. After three minutes of rest following the last MVC, the first sEMG signal (EMG-1) was collected. The volunteer was instructed to perform a “step contraction” consisting of four force levels (15, 20, 25, and 30% MVC). Contraction time was 11 seconds for each force level. Feedback of the step contraction was provided from the projection of a 20′′ screen on a white wall in front of the subject. All participants received training prior to the shoulder elevations based on the previously determined force levels.

After one minute of rest EMG-1, the NRS was used to assess pain intensity in the NPG (pre-AA). AA will then be performed, with the needles remaining inserted for 30 minutes. After the removal of the needles, further evaluations of pain were performed (post-AA), followed by a second EMG reading (EMG-2) in the same manner as performed during EMG-1.

### 2.6. EMG Signal Processing

For the analysis of sEMG signal, the first second of each step (considered a transition time between force levels) was discarded. The root mean square (RMS) was calculated using 200 ms moving window. The EMG signals were processed using specific routines carried out in the Matlab program, R2010b (The MathWorks Inc., Natick, MA, USA).

### 2.7. Data Analysis

The Shapiro-Wilk test demonstrated that the data were normally distributed. The independent *t*-test was used to compare the means between health subjects and NS-NP patients. The two-way repeated-measures (ANOVA) design was used to analyze the influence of AA treatment on EMG activity of the upper trapezius muscle and pain (NRS). Specific differences were determined from a post hoc analysis using a Bonferroni adjusted test and *t*-test for multiple pairwise comparisons while maintaining an alpha level of 0.05 for significance in each of the analyses. All data were analyzed using SPSS 20.0 software (SPSS Inc., Chicago, USA).

## 3. Results

Table 1 presents health subjects and NS-NP patients demographic data. There were no significant differences between the groups with respect to demographic data, age, and weight (*p* > 0.05).

When analyzing the effects of the intervention with AA and SAA on pain intensity (NRS) in the NS-NP group, ANOVA confirmed a significant effect for the treatment (before* versus* after) (*F* = 48.40; *p* < 0.0001), regardless of the type of intervention (treatment* versus *groups) (*F* = 0.40; *p* = 0.53). The pre- and posttreatment comparisons can be seen in Table 2.

**Figure 3:**
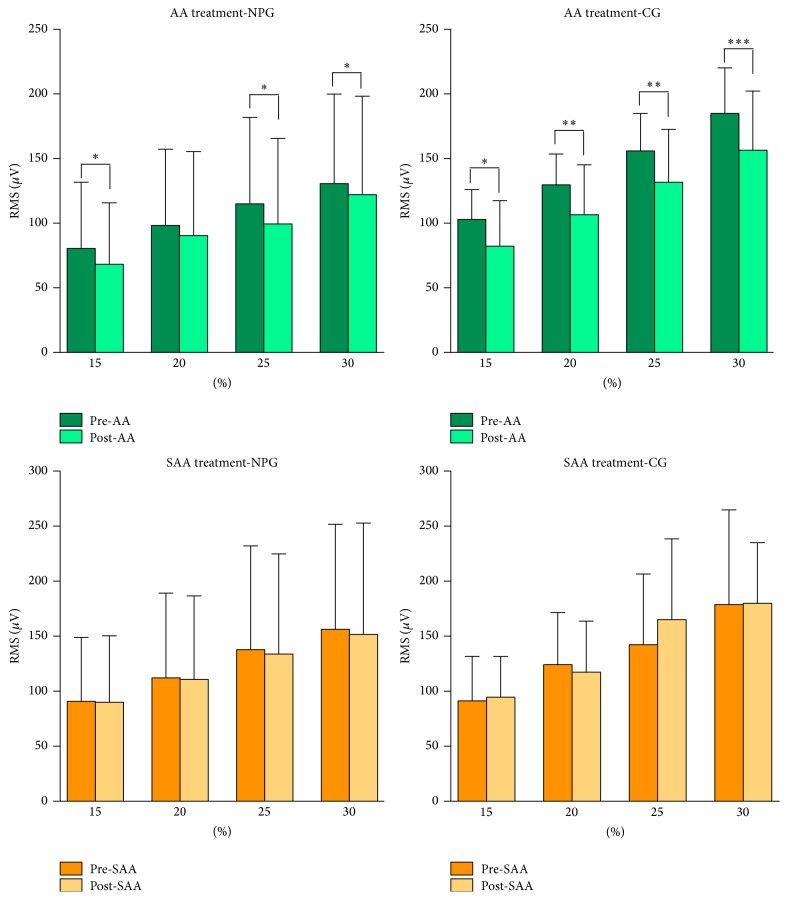
Mean and standard deviation of the RMS obtained before and after treatment with Nogier's auricular acupuncture (AA) and sham auricular acupuncture (SAA) in individuals with nonspecific neck pain (NPG) and in healthy individuals (CG). (^*∗*^ *p* < 0.05; ^*∗∗*^ *p* < 0.001; ^*∗∗∗*^ *p* < 0.0001: unpaired *t*-test.)

With regard to the EMG activity, it was possible to observe a significant decrease in the RMS for both groups treated with AA (NS-NP group: *F* = 18.10, *p* < 0.0001; HS group: *F* = 51.36, *p* < 0.0001; ANOVA test). These differences were not observed for the SAA treatment (NS-NP group: *F* = 0.73, *p* = 0.39; HS group: *F* = 1.22, *p* < 0.27). The mean and standard deviation values, as well as significant differences in the RMS value before and after treatment, can be seen in Figure 3.

## 4. Discussion

AA is a form of alternative treatment based on the idea that all of the body parts are represented in locations in the skin of the external auricle. This treatment method has been applied in order to provide pain relief, relaxation, and other effects [30–33]. It has also been used as an adjunct therapy for pain management, thereby reducing the use of analgesics and minimizing potential adverse effects and tolerance [21, 30].

However, in this study, the effects of AA on the pain assessed in the NRS were practically the same as those observed in the SAA. This result indicates that the decrease in cervical pain found in the present study for the two types of intervention (AA and SAA) is more closely associated with the peripheral stimulus provided by the insertion of the needle than with specific points indicated for the treatment of neck pain [22, 23]. This hypothesis is based on the fact that the cutaneous penetration of the needles always causes a physiological reaction, for example, the triggering of neural pathways, resulting in diffuse noxious inhibitory control [34].

The auricular point used in SAA is another factor that might have influenced the results in this study. Although this region does not present any somatotopic relationship to the shoulder and the scapular waist according to Nogier theory [23], this point is localized at the region of auricular concha which is mainly innervated by auricular branch of vagus nerve and a potential analgesic effect was observed after transcutaneous stimulation in this region [35]. Thus, this observation should be considered in future studies.

Furthermore, the findings of this study are similar to the conclusion of two systematic reviews, with meta-analysis, of the control of pain using traditional acupuncture. In these studies, it was concluded that the differences found between true and sham acupuncture were relatively modest, with a mild analgesic effect that seems to lack clinical relevance and cannot be clearly distinguished from bias [36, 37]. Given this preliminary evidence, the specificity of auricular points for the treatment of NS-NP should be tested and confirmed in larger controlled studies.

Conversely, the AA treatment significantly decreased the EMG activity of the upper trapezius muscle in the two groups studied (NS-NP and HS), whereas there was almost no change in the EMG signal recorded after treatment with SAA. EMG evidence of abnormal upper trapezius muscle activity after the insertion of needles in the same points used in the present study has also been put forth in previous studies [24, 38]. Therefore, these results could provide a better understanding of the physiological mechanisms of AA.

In general, the most common methods of quantifying pain (the numerical rating scale and the visual analogue scale) are subjective. Consequently, it is possible that the patient's expectations of treatment outcomes could have a psychological impact that may affect the scores of these indices. On the other hand, when the EMG signal is collected with submaximal isometric contractions, it is less likely to be affected by the expectations of the patient and thereby provides a more objective assessment of the neurophysiological conditions of the muscle studied.

The decrease of pain in both treatments (AA and SAA), as well as in EMG activity for individuals treated with AA, also reinforces the possibility that the results found in the SAA treatment could have been affected by a certain level of psychological expectation on behalf of the patient. However, these arguments must be addressed with great care. Generally, muscle pain induces a decrease in the net excitatory input to the motor neuron pool innervating the painful muscle, which causes reduced muscle activity [39]. If this same relationship was considered in terms of the results of the present study, the EMG signal should have increased in relation to the decrease in pain found after the AA treatment.

However, the fact that the AA treatment decreased the EMG activity of the upper trapezius muscle in NS-NP and HS group indicates that the stimulus in the acupoints involves a neurophysiological action that goes beyond pain inhibition. In this case, the stimulus provided by the AA leads to the generation of a mechanism that inhibits muscular activity. Although this hypothesis requires further research to be confirmed, this physiological effect could contribute to muscle relaxation and a consequent clinical improvement in the patient. This observation is a relevant factor that should be investigated in future studies.

The effects of AA on the EMG activity of the upper trapezius muscle in the present study provide scientific evidence that supports Nogier's theory, which defends the idea of somatotopic correspondence between auricular points and specific (i.e., projected) body areas [22, 23].

Finally, it is important to note that the results of the present study refer to the effects observed after a single session of treatment with AA. To our knowledge, there has been no randomized controlled trial assessing the effectiveness of AA as a complementary therapy for the relief of acute pain or the improvement of cervical function in NS-NP patients. Therefore, the present study provides the basis for the performance of new studies to assess the efficiency of NS-NP with AA, as suggested by Nogier method [22, 23].

The small sample size and the point used for treatment with SAA can be considered as main limitations of this study. Although the sample size has been previously calculated, the improvement in pain as one of the possible outcomes was not considered. The potential analgesic effects in the region in which the point is located and used for treatment with SAA [35] also limit the ability to draw definitive conclusions from the data obtained in this study.

## 5. Conclusion

This study demonstrated the immediate effect of auricular acupuncture (Nogier method) on the electromyographic activity of the upper trapezius muscle in patients with nonspecific neck pain and healthy subjects. The effect of this intervention on pain symptoms in patients with NSNP was inconclusive, given that the decreases observed in the true and sham auricular acupuncture treatment protocols were practically the same, suggesting that further investigation is necessary.

## Figures and Tables

**Table 1:** Demographic data of patients (mean ± SD).

	Health subjects	NS-NP patients	*p* value^*∗*^
Age (year)	24.42 ± 1.90	22.89 ± 2.63	0.89
Sex (M/F)	3/9	3/9	—
Weight (Kg)	57.72 ± 4.11	59.72 ± 3.16	0.23

^*∗*^Independent *t-*test.

**Table 2:** Mean and standard deviation (±SD) of pain intensity obtained by the NRS.

Treatment	Before	After	Mean difference	*p* value^*∗*^
AA	4.25 ± 1.13	2.25 ± 0.97	2.00 ± 0.16	*p* < 0.0001
SAA	4.00 ± 0.73	2.33 ± 1.07	1.67 ± 0.34	*p* < 0.0001

AA: auricular acupuncture treatment. SAA: sham auricular acupuncture treatment.

^*∗*^ *t*-test (post hoc test).
